# Association between Macronutrients Intake, Visceral Obesity and Blood Pressure in a Sample of Obese Egyptian Women

**DOI:** 10.3889/oamjms.2015.029

**Published:** 2015-02-26

**Authors:** Nayera E. Hassan, Salwa M. El Shebini, Nihad H. Ahmed, Mohamed Selim Mostafa

**Affiliations:** 1 *Biological Anthropology Department, National Research Centre, Cairo, Egypt*; 2 *Nutrition and Food Science Department, National Research Centre, Cairo, Egypt*; 3 *Researches and Applications of Complementary Medicine Department, National Research Centre, Giza, Cairo, Egypt (Affiliation ID: 60014618)*

**Keywords:** Blood pressure, macronutrients, obesity, visceral obesity

## Abstract

**AIM:**

Study the association between the total caloric intake, protein, lipid, and some classes of fatty acids of the diet, and their effects on blood pressure in a sample of Egyptian obese women with and without visceral obesity.

**METHODS:**

Five hundred forty-nine obese women were included in the study with mean age of 38.1 ± 11.56 years and mean Body mass index [BMI] of 36.17 ± 7.23. They enrolled in a program for losing weight. Visceral fat was determined using ultrasound. Blood pressure was measured 3 times and the mean was recorded. Twenty four hours dietary recall was reported.

**RESULTS:**

Thirty point four percentages of samples has visceral obesity ≥ 7cm; they were the older, showed higher values of BMI, visceral obesity and blood pressure. Significant difference was found between groups regarding mean value of BMI, visceral obesity, both systolic blood pressure SBP and diastolic blood pressure DBP and most of the daily macronutrients intake. In groups (2&3) positive significant correlation was recorded between (SBP) & (DBP) and total daily intake of total calories, carbohydrate, total fat, saturated fatty acids and cholesterol, and negative significant correlation with total daily intake of total protein, animal and vegetable protein, linolenic and linoleic fatty acids, while oleic fatty acid showed negative correlation with SBP&DBP in all groups.

**CONCLUSION:**

This study emphasizes the hypothesis that the macronutrients composition of diet influences blood pressure in different ways, in obese patients with visceral obesity.

## Introduction

Elevated blood pressure (BP) continues to be a major public health concern because of its high global prevalence and its concomitant increase as risk of cardiovascular disease [1, 2]. Studies have shown that small decreases in population average systolic blood pressure (SBP) or diastolic blood pressure (DBP) could reduce the burden of BP-related diseases [3-5]. Lifestyle modifications such as losing weight, increasing physical activity, and reducing consumption of alcohol and salt have been consistently shown to decrease [5].

Energy balance and macronutrient balance are the cornerstones upon which any theories of obesity must be built. Obesity can only occur when energy intake remains higher than energy expenditure for an extended period of time. However the macronutrient composition of the diet can also affect energy balance. Fat is a key nutrient because it is poorly regulated at both the level of consumption and oxidation [6]. Dietary fat accelerates renal injury and peripheral vascular dysfunction and promotes visceral obesity in a disease model with chronic inflammation [7].

Saturated fatty fats (SFAs) have a bad file and several experimental studies in the rat showed a progressive increase in blood pressure in response to a highly saturated diet [8]. Moreover, a highly saturated diet during gestation led to offspring which, when adults, presented a gender-related hypertension. The mechanism of this effect may be related to the polyunsaturated/saturated ratio (p/s). Polyunsaturated fatty acids (PUSFAs) have been shown to exert a positive action on hypertension. This effect could be attributed to the alteration of the p/s, but mainly to the omega3 polyunsaturated fatty acids. The comparison of several animal models led to the conclusion that long-chain omega3 can prevent the increase in blood pressure and reduce established hypertension [8].

Little is known about the relation of different dietary protein types with blood pressure. Altorf-Van [9], reported that total protein and animal protein were not associated with BP in general untreated Dutch population. Plant protein may be beneficial to BP, especially in people with elevated BP because high intake of plant protein may be a marker of a healthy diet and lifestyle in general, confirmation from randomized controlled trials is warranted. Predominantly vegetarian diet has been associated with lower BP; the difference is more likely to be due to lifestyle factors common to vegetarians and to the types and amounts of fats in vegetarian diets rather than an inherent benefit of vegetable source of protein [9].

The aim of this study focuses on the quantitative concern of total caloric, protein, lipid, and some classes of fatty acids of the lipid fraction of the diet, and their effects on blood pressure in a sample of Egyptian obese women with and without visceral obesity.

## Subjects and Methods

Five hundred forty nine obese women, shared as volunteers in this study. They were enrolled in a program for losing weight in National Research Centre (NRC) Egypt that lasted for 24 month, after taking approval from Ethical Committee of NRC and written informed consent from each of them. Their mean age was 38.1 ± 11.56 years and had a mean BMI of 36.17 ± 7.23 kg/m^2^. All women were subjected to thorough clinical examination

### Anthropometric parameters and blood pressure measurements

Relevant anthropometric measurements were reported including height and weight using standardized equipment, and following the recommendations of the International Biological Program [10]. Body mass index (BMI) was calculated (weight in kg/height in meter^2^). All participants had BMI >29 kg/m^2^.

Blood pressure for each of them was measured 3 times in the same setting and the mean value was recorded. Systolic and diastolic blood pressures were measured throughout for each subject in the sitting position using standardized mercury sphygmomanometer with an appropriate blood pressure cuff that covers at least two thirds of the right upper arm length and does not encroach on the antecubital space.

Visceral fat was determined by using ultra sound device. Ultrasound (US) examination to each participant was done to evaluate visceral fat at the umbilicus (USVF) in cm. Intrabdominal fat thickness measurement was obtained using the “Medison Sonoace X8” ultrasonography equipment. For the visceral fat, a 3.5 MHz transducer was transversely positioned 1 cm above the umbilical scar on the abdominal midline, without exerting any pressure over the abdomen. Visceral fat thickness attempted corresponding to the measurement in centimeters between the internal surface of the abdominal rectus muscle and the posterior aortic wall in the abdominal midline, during expiration. Cutoff point of umbilical visceral fat thickness ≥7 cm was assessed for diagnosis of visceral obesity [11]. In the current research, we considered that visceral fat thickness < 3 cm (normal), 3-< 7(Border line) grade I visceral obesity; ≥ 7 cm grade 2 visceral obesity [11]. Then, the sample was classified according to visceral fat thickness to 3 groups: group (1): visceral fat thickness <3 cm, group (2): visceral fat thickness 3-<7 cm, group (3): visceral fat thickness> 7 cm.

### Dietary recalls

Collecting detailed data about food intake was done through 24 hour recall that repeated for 3 days. Analysis of food items was done using World Food Dietary Assessment System, (WFDAS), 1995, USA, University of California.

### Statistical analysis

All values were expressed as mean value ± SD. Two tailed student t-test was used to compare between the two groups. Correlation between the different parameters was tested by Pearson test. P values <0.05 were considered statistically significant. SPSS windows software version 17.0 (SPSS Inc. Chicago, IL, USA, 2008) was used.

## Results

Sample distribution, according to the degree of visceral obesity (VO) presented in Table 1 shows that 30.4% of the sample proved to have the higher degree of VO (≥7 cm), group (3) while only 6.2% had <3cm degree, group (1), the rest (63.4%) were between 3-<7cm, group (2).

**Table 1:** Distribution of the sample according to degree of visceral obesity.

Groups	Group1 (< 3)	Group2 (3 - <7)	Group3 (≥7)	Total
Number	34	348	167	549

Percent (%)	6.2	63.4	30.4	100

Group1:< 3 Normal Group2: 3- < 7 Border line Group3: ≥ 7 Visceral obese

Table 2 shows the mean ±SD of the age, BMI, visceral obesity and blood pressure among the obese women according to the degree of visceral obesity. Group (3) was the older, showed the higher values of BMI, visceral obesity and blood pressure. Significant difference was found between group (1) and groups (2), and between group (2) and group (3) regarding the mean value BMI, VO and both SBP and DBP.

**Table 2:** Mean ± SD of BMI, Visceral obesity and blood pressure among the obese women according to the different groups.

Groups	Group 1 (< 3)	Group 2 (3 - <7)	Group 3 (≥7)
Age (yrs)	36.41 ± 11.02	38.40 ± 12.04	39.51 ± 11.50
BMI (Kg/m^2^)	29.45 ± 4.65	34.36 ± 5.99^a^	42.05 ± 6.83^b^
Visceral obesity	2.31 ± 0.62	5.04 ± 1.09^a^	8.55 ± 1.51
S B P (mmHg)	121.91 ± 18.34	121.56 ± 19.9^a^	132.19 ± 19.97^b^
D B P (mmHg)	79.97 ± 12.11	79.97 ± 12.82^a^	85.95 ± 15.57^b^

a: Group1 vs. Group 2; b: Group 1 vs. Group 3; Significant at p < 0.001; BMI = Body mass index; SBP = Systolic blood pressure; DBP = Diastolic blood pressure.

Means ± SD per daily intake of the different macronutrients of the different groups presented in Table 3. Group (3) had the higher consumption level of the all recorded nutrients. Significant difference was found between group (1) and group (2) and between group (2) and group (3) as regard animal protein, SFAs and the PUFAs, linolenic acid, while significant difference was found between group (2) and group (3) for total energy, vegetable protein, the monounsaturated fatty acid (MUFAs), oleic acid, and the PUSFA linoleic acid.

**Table 3:** Mean ± SD & % Recommended Dietary Allowances (RDAs) of the macronutrient intake/day among the obese women according to the different groups.

Visceral	Group 1 (<3)	Group 2 (3 -7)	Group 3 (≥7)
Nutrient intake	Mean± S D %RDAs	Mean± S D % RDAs	Mean ± S D % RDAs	RDA
Energy (Kcal)	2600.14 ± 524.03 118.19	2641.93 ± 731.34 120.09	2728.11 ± 699.89^b^ 124.01	2200
Total Protein (g)	91.34 ± 25.30 182.68	90.90 ± 26.11 181.80	92.37 ± 25.46 184.74	50
Vegetable protein (g)	53.73 ± 31.05	48.93 ± 28.25	42.43 ± 32.24^b^	
Animal protein (g)	37.61 ± 22.35	41.9 7 ± 0.41^a^	49.94 ± 24.52^b^	
Total Fat (g)	119.77 ± 38.38	120.20 ± 40.44	123.36 ± 90.55	
Saturated fatty acids (g)	53.01 ± 13.12	72.14 ± 14.32^a^	80.87 ± 10.56^b^	
Unsaturated fatty acids (g)	66.76 ± 14.25	48.06 ± 13.11	42.49 ± 17.15^b^	
Cholesterol (mg)	332.86 ± 186.06	413.93 ± 279.37	415.86 ± 247.85	
Oleic acid (g)	26.03 ± 8.12 38.99*	15.98 ± 4.20 33.25*	13.76 ± 3.64^b^ 32.09*	
Linolenic acid (g)	11.43 ± 2.24 17.12*	6.23 ± 2.72^a^ 12.96*	5.39 ± 2.92^b^ 12.57*	
Linoleic acid (g)	13.76 ± 5.19 20.61*	10.76 ± 3.25 22.38*	9.45 ± 4.75^b^ 22.24*	
Carbohydrate (g)	267.08 ± 76.30	293.27 ± 84.84	304.27 ± 84.85	
Linoleic/linolenic ratio	1.20	1.73	1.75	
P/U ratio	1.26	0.67	0.53	

a: Group1 vs Group 2; b: Group 1 vs Group 3; Significant at P<0.05;

* Percent of unsaturated fatty acids.

Tables 4 & 5 show the correlation coefficient between the daily intake of the different macronutrients and the systolic and diastolic blood pressure in the different three groups. High positive significant correlation was detected between SBP&DBP and the daily total caloric intake, carbohydrate, total fat, SFAs, and cholesterol in Group (2&3). Significant negative correlation was found between the daily total protein, animal, vegetable protein, linolenic and linoleic fatty acids intake and SBP&DBP, in the same groups, while significant negative correlation was detected between the oleic fatty acid and both SBP&DBP in the three groups.

**Table 4:** Correlation coefficient between the daily intakes of macronutrients and blood pressure among obese women in the three groups.

Items	Group1 (<3)		Group 2 (3-7)		Group3 ((≥7)	
Blood pressure (mmHg)	S B P	D B P	S B P	D B P	S B P	D B P
Energy (Kcal)	0.284	0.294	0.361**	0.284**	0.362**	0.421**
Protein (g)	0.157	0.169	-0.573**	-0.477**	-0.238^**^	-0.211**
Vegetable protein (g)	0.271	0.110	-0.409**	-0.270**	-0.507**	-0.331**
Animal protein (g)	0.140	0.107	-0.201**	-0.029	-0.191*	-0.200*
Total CHO (g)	0.130	0.149	0.166*	0.172*	0.183*	0.170*

CHO: Carbohydrates;

* Significant at P < 0.05;

** Significant at P < 0.01.

**Table 5:** Correlation coefficient between total fat intake, different fatty acids and blood pressure among the obese women in the different groups.

Items	Group 1 (<3)		Group 2 (3-7)		Group 3 ((≥7)	
Blood pressure (mmHg)	S B P	D B P	S B P	D B P	S B P	D B P
Total Fat (g)	0.136	0.125	0.457**	0.315**	0.351**	0.242**
Saturated fatty acids (g)	0.284	0.175	0.126*	0.495**	0.320**	0.241**
Unsaturated fatty acids (g)	-0.210	-0.243	-0.101	-0.249**	-0.371**	-0.277**
Cholesterol (mg)	0.175	0.214	0.421**	0.410**	0.327**	0.390**
Oleic acid (mg)	-0.375*	-0.681*	-0.477**	-0.320**	-0.244**	-0.433**
Linolenic acid (mg)	-0.103	-0.200	-0.355**	-0.271**	-0.485**	-0.479**
Linoleic acid (mg)	-0.110	-0.158	-0.177*	-0.214**	-0.281**	-0.250**

* Significant at P < 0.05;

** Significant at P < 0.01.

## Discussion

Data of this study revealed that overweight and obese women in the different three groups consumed high amount of calories, protein and fat in their daily diet compared to the RDAs, where it reached 124.01% and 184.74% of the RDAs for the mean daily caloric and protein intake respectively in group (3).

Positive significant correlations were found between both SBP and DBP and the mean daily intake of total calories, carbohydrate, total fat, SFAs, and cholesterol in groups (2&3) that had higher degree of BMI and visceral obesity, which is in line with that reported by Najafian [12], who stated that the usual relation between caloric intake and blood pressure in obese may be due to insulin resistance induced by obesity, as total daily caloric intake in general population had no significant effect on blood pressure and on development of hypertension when the effect of obesity is adjusted.

On the other hand, negative significant correlations were found between SBP & DBP and the daily intake of the total protein, vegetable and the animal protein in groups (2&3). Friedman et al [13], observed significant BP reductions associated with increased consumption of both animal protein and vegetable protein, with no heterogeneity in BP reduction based on protein source. These findings have important public health and clinical implications, suggesting that replacement of carbohydrate intake with protein intake could be an important strategy for helping to curb the growing pandemic of hypertension and related cardiovascular disease morbidity and mortality [13]. Several lines of evidence support an independent association of protein with BP. Protein, having a lower glycemic index than carbohydrate, produces smaller increases in glucose and insulin. The hyperinsulinemic state has been posited as a pathophysiologic mechanism underlying hypertension development and could help to explain these findings [14, 15]; these reports suggested that there could be cross-talk among the visceral fat depot, the renin-angiotensin system, and insulin resistance in promoting the regulation of blood pressure. The Dash (Dietary Approaches to Stop Hypertension) diet, a widely used intervention for BP reduction, includes increasing ones intake of protein-rich founds to 18% of total energy compared with the average protein intake of 15.2% of total energy in United States, (Dietary Guidelines Advisory Committee, 2010), [16]. In addition, amino acids in proteins have been shown to lower BP, [17] Dietary protein intake may also increase levels of tryptophan and tyrosine, which have been shown to reduce blood pressure in animal experiments [18, 19]. There is also evidence that amino acids may act directly on the kidney to affect BP through increased renal plasma flow, glomerular filtration rate, and sodium excretion [19]. On the other hand Tielemans et al., [20] reported that intake of plant protein, but not animal protein, was inversely associated with 5-year changes in BP level in elderly men.

Observational studies indicate that dietary saturated fat is positively associated with blood pressure [21, 22], and polyunsaturated fat and the polyunsaturated/saturated fat (P/S) ratio are inversely associated with blood pressure [21, 23], that is in line with our results where the lowest ratio was detected in group (3). However, contradict reports that found no such association was reported by Ascherio et al [24]. Positive significant correlation was detected in this study mainly in groups (2&3) between dietary cholesterol and both SBP and DBP. Sakurai et al [25], found a low-order, positive relationship of dietary cholesterol intake to SBP with control for multiple possible confounders, however reduction of dietary cholesterol intake may contribute to prevention and control of adverse blood pressure levels in general populations.

The role of individual fatty acids in blood pressure regulation is unclear. Data of this study revealed high negative significant correlation between the monounsaturated fatty acids (MUFA), the oleic fatty acid and both SBP and DBP in the overweight women (group 1) and in the same time obese women who had BMI ≥30 and visceral obesity that support its important healthy effect in control of BP level. Mediterranean dietary pattern has been proposed as a healthy choice for the prevention of cardiovascular disease, part of its beneficial impact can be mediated through a favorable effect on blood pressure (BP). A major characteristic of the Mediterranean diet is a high supply of energy coming from MUFA, [26]. Increase oleic fatty acid levels in membranes, regulates membrane lipid structure (H(II) phase propensity) in such a way as to control G protein-mediated signaling, causing a reduction in BP. This effect is in part caused by its regulatory action on G protein-associated cascades that regulate adenylyl cyclase and phospholipase C [27]. The International Study of Macro/Micronutrients and Blood Pressure is a cross-sectional epidemiologic study of 4680 men and women ages 40-59 years from 17 population samples, reported an inverse associations of dietary total oleic acid (main MUFAs) with blood pressure in those obtained oleic acid from vegetable sources. In addition they stated that dietary linoleic acid intake may contribute to prevention and control of adverse blood pressure levels in general populations [28], which in line with our results. In this setting Appel et al [29], reported that a healthful diet, partial substitution of carbohydrate with either protein or monounsaturated fat can further lower blood pressure, improve lipid levels, and reduce estimated cardiovascular risk. However, higher-protein diets probably improve adiposity, blood pressure and triglyceride levels, but these effects are small and need to be weighed against the potential for harms [30].

In conclusion, consumption of healthy diet characterized by balanced macronutrients, protein, fat and carbohydrate, rich in monounsaturated, polyunsaturated fatty acids may contribute to prevention and control of adverse blood pressure levels.
